# Literary Notes

**Published:** 1910-02

**Authors:** 


### LITERARY NOTES

"HUDSON-FULTON Celebration 1609-1807-1909," is the title of a handsome brochure compiled and edited by Harlan Hoyt Horner, Esq., in commemoration of the Hudson-Fulton Celebration, September 25 to October 9, 1909. It is intended as a manual for the use of the schools of the state, for which purpose it was sent out last fall by the State Education Department. The Commissioner of Education, Dr. Andrew S. Draper, writes an article for the book, entitled "The Hudson-Fulton Celebration," which very properly opens the series and, after giving something of history relating to Hudson river, urges pupils to read and write upon the history which makes for the greatness of the Empire State. Other articles relate to the plans of the Commission; the observance in the schools; Hudson and the River, the latter by Charles Elliott Fitch; Fulton and the Clermont, poems and extracts in verse, bibliography, and much other interesting and instructive material. It is handsomely printed on heavy calendered and tinted paper, beautifully illustrated, and constitutes a valuable contribution to the history of an event which thus will be fittingly preserved in an enduring form. It is a credit to the education department as well as to the editor and those who contributed to its pages.

